# Effects of Arachidonic and Docosohexahenoic Acid Supplementation during Gestation in Rats. Implication of Placental Oxidative Stress

**DOI:** 10.3390/ijms19123863

**Published:** 2018-12-04

**Authors:** Cynthia Guadalupe Reyes-Hernández, David Ramiro-Cortijo, Pilar Rodríguez-Rodríguez, Sonia Giambelluca, Manuela Simonato, Mª del Carmen González, Angel Luis López de Pablo, Mª del Rosario López-Giménez, Paola Cogo, Miguel Sáenz de Pipaón, Virgilio P. Carnielli, Silvia M. Arribas

**Affiliations:** 1 Department of Physiology, Faculty of Medicine, University Autónoma of Madrid, 28029 Madrid, Spain; cynthia.grh3@yahoo.com (C.G.R.-H.); david.ramiro@uam.es (D.R.-C.); pilar.rodriguezr@uam.es (P.R.-R.); m.c.gonzalez@uam.es (M.d.C.G.); angel.lopezdepablo@uam.es (A.L.L.d.P.); 2 Fondazione Istituto di Ricerca Pediatrica Cittá della Speranza, 35129 Padova, Italy; sonia.giambelluca@gmail.com (S.G.); lab2nut@gmail.com (M.S.); 3 Department of Epidemiology, Public Health & Microbiology, Faculty of Medicine, University Autónoma of Madrid, 28029 Madrid, Spain; mrosario.lopez@uam.es; 4 Division of Pediatrics, Department of Medicine, University of Udine, 33100 Udine, Italy; paola.cogo@uniud.it; 5 Department of Neonatology, La Paz Hospital-University Autónoma of Madrid, 28046 Madrid, Spain; miguel.saenz@salud.madrid.org; 6 Carlos III Health Institute, Maternal and Child Health and Development Research Network, 28029 Madrid, Spain; 7 Division of Neonatology, Department of Clinical Sciences, Polytechnic University of Marche, 60121 Ancona, Italy; v.carnielli@staff.univpm.it; 8 Azienda Ospedaliero, Universitaria Ospedali Riuniti, 60030 Ancona, Italy

**Keywords:** ARA, DHA, oxidative stress, IUGR, fetus, placenta

## Abstract

Arachidonic and docosahexaenoic acids (ARA and DHA) are important during pregnancy. However, the effects of dietary supplementation on fetal growth and oxidative stress are inconclusive. We aimed to assess the effect of high ARA and DHA diet during rat gestation on: (1) ARA and DHA availability in plasma and placenta, (2) fetal growth, and (3) placental oxidative stress, analyzing the influence of sex. Experimental diet (ED) was prepared by substituting soybean oil in the control diet (CD) by a fungi/algae-based oil containing ARA and DHA (2:1). Rats were fed with CD or ED during gestation; plasma, placenta, and fetuses were obtained at gestational day 20. DHA, ARA, and their precursors were analyzed in maternal plasma and placenta by gas chromatography/mass spectrophotometry. Fetuses and placentas were weighed, the proportion of fetuses with intrauterine growth restriction (IUGR) determined, and placental lipid and protein oxidation analyzed. ED fetuses exhibited lower body weight compared to CD, being >40% IUGR; fetal weight negatively correlated with maternal plasma ARA, but not DHA. Only ED female placenta exhibited higher lipid and protein oxidation compared to its CD counterparts; lipid peroxidation is negatively associated with fetal weight. In conclusion, high ARA during gestation associates with IUGR, through placental oxidative stress, with females being more susceptible.

## 1. Introduction

A maternal diet is critical for a successful pregnancy, as well as for fetal health [1,2]. Fatty acids (FA) are important biological constituents, with metabolic, structural, and signaling roles, it is well established that the quantity and quality of dietary fats during pregnancy have relevant effects for the mother and offspring health [3,4]. Linoleic acid (LA; 18:2n-6) and α-linolenic acid (ALA; 18:3n-3) are the essential fatty acids from which long-chain polyunsaturated fatty acids (LCPUFAs) can be synthesized. In the human fetus the most important n-3 and n-6 LCPUFAs are eicosapentaenoic acid (EPA; 20:5n-3), docosahexaenoic acid (DHA; 22:6n-3), and arachidonic acid (ARA; 20:4n-6) [5]. The fetus has a limited capacity to carry out the synthesis from their precursors LA and ALA, due to limited enzyme availability, and they are most readily obtained from the maternal dietary intake and metabolism [6,7]. These FA access the fetus through placental transfer [4], primarily in the last trimester of pregnancy. Western diets usually exhibit a disequilibrium between n-3 and n-6 FA, with a larger proportion of the latter [8]. Therefore, it has been proposed that supplementation during gestation should be based on n-3 FA. However, in humans, the benefits of this supplementation remain inconclusive, as shown by several systematic reviews [9,10], and more studies are needed to ascertain the consequences of supplementation with n-3 and n-6 LCPUFAs during pregnancy and their role for fetal growth [11]. This question also remains controversial in rodents. In mice, there is evidence that dams supplemented with n-3 LCPUFA tend to produce smaller litters; their pups suckle less and gain less weight compared to those supplemented with n-6 series [12]. In rats, moderate amounts of fish oil supplementation during pregnancy do not improve growth, but instead lead to intrauterine growth restriction (IUGR) [13]. Both ARA and DHA are needed for fetal development, and a deficiency in one may compromise growth [14]. It has been shown that growth deficiency induced by fish oil supplementation is related to a reduced ARA availability due to inhibition of its synthesis by the excess of DHA [15].

Another important aspect of n-3 and n-6 LCPUFAs is their role in oxidative stress and in inflammatory responses. This is particularly relevant during pregnancy, where oxidative stress has been implicated in the development of materno-fetal complications such IUGR [16]. LCPUFAs of the n-3 series exert anti-inflammatory properties, while ARA-derived eicosanoids, such as prostaglandins and leukotrienes, contribute to inflammation [17]. The role of n-3 PUFAs on oxidative stress also remains controversial. On one hand, n-3 LCPUFAs are highly susceptible to lipid peroxidation and there is evidence that supplementation may increase Reactive Oxygen Species (ROS) production and cellular damage [18]. On the other hand, a ROS scavenger action of n-3, but not n-6 FA, has been shown in vitro [19] and it has been suggested that supplementation with n-3 LC-PUFAs may limit oxidative stress [20]. In pregnancy, the effect of n-3 LC-PUFA supplementation on placental oxidative stress and inflammation seems to be dependent on the dose and timing and further studies are needed to answer these questions [21].

Our aim was to shed light on some of these aspects. We evaluated the effect of a diet with a high ARA and DHA content, given during gestation to Sprague Dawley rats, assessing if the supplemented diet: (1) Increase the availability of ARA and DHA in maternal plasma and placenta; and (2) is beneficial or detrimental for fetal growth, evaluating the role of placental oxidative stress. We have also analyzed the possible influence of sex on the responses to the dietary intervention.

## 2. Results

### 2.1. Diet Intake

A group of experiments was performed to evaluate the amount of chow the rats consumed during gestation. We did not detect significant differences in the amount of daily chow between CD-fed and ED-fed dams (CD = 61.8 ± 4.1 g/kg/day, *n* = 6; ED = 59.3 ± 2.2 g/kg/day, *n* = 6; *p* = 0.63; and Student´s *t* test). The calculated amount of DHA and ARA ingested by rats fed ED was 164.6 mg/kg/day and 349.5 mg/kg/day, respectively. The LCPUFA were not present in the CD diet.

### 2.2. Maternal Plasma Composition

We did not detect statistical differences between CD and ED groups neither in maternal plasma protein (CD = 82.1 ± 11.6 mg/mL; ED = 78.9 ± 7.7 mg/mL; *p* = 0.74; and Student’s *t* test) nor in glucose concentrations (CD = 104.5 ± 16.2 mg/dL; ED = 107.0 ± 8.8 mg/dL; *p* = 0.96; and Student’s *t* test).

**Figure 1:**
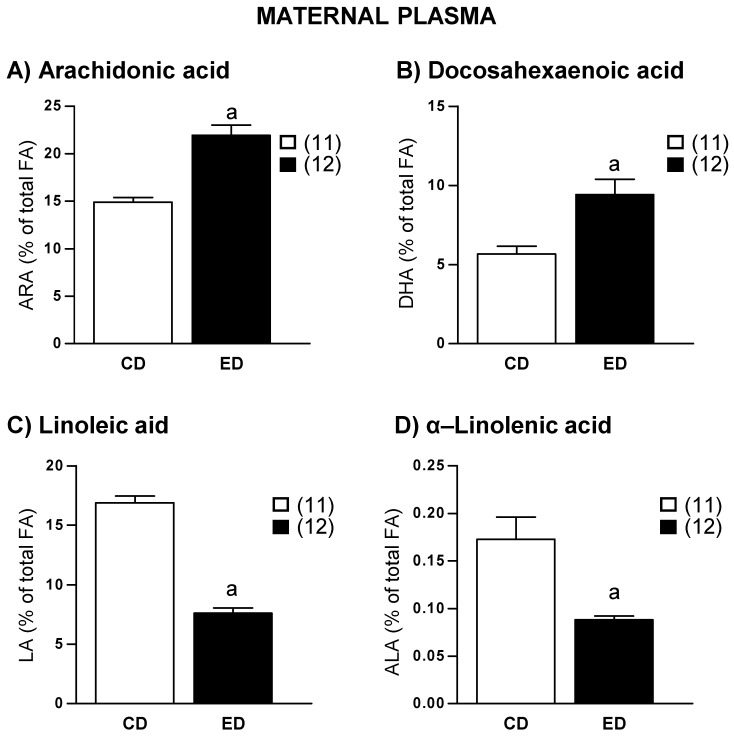
Plasma levels of (**A**) Arachidonic acid (ARA), (**B**) Docosahexaenoic acid (DHA), (**C**) Linoleic acid (LA), and (**D**) α-Linolenic acid (ALA) in dams fed with control diet (CD) and experimental diet (ED). Data show mean ± SEM. Between parenthesis, the sample size is shown. Student’s *t* test. ^a^ *p* < 0.001 ED versus CD.

Plasma ARA, DHA, and their precursors were analyzed in the phospholipid, triglyceride, and free FA fractions. Since we obtained similar results in all the fractions, we only present the data from the phospholipid fraction, which is the most representative in plasma. ARA and DHA levels were significantly higher in ED compared to CD dams (Figure 1A,B). Instead, the concentration of the precursors LA and ALA were significantly lower in ED compared to CD dams (Figure 1C,D). A negative correlation was found between ARA and DHA plasma levels (*r* = −0.94, *r*^2^ = 0.88, and *p* = 0.0001).

### 2.3. Gestation Outcome

At the end of gestation ED dams had a significantly lower body weight compared to CD dams (CD = 447 ± 39 g, *n* = 13; ED = 401 ± 33 g, *n* = 9; *p* = 0.0001; and Student’s *t* test). However, the weight of the dam without conceptus was similar in both groups (CD = 369 ± 46 g, *n* = 12, ED = 355 ± 17 g, *n* = 9; *p* = 0.38; and Student’s *t* test).

**Figure 2:**
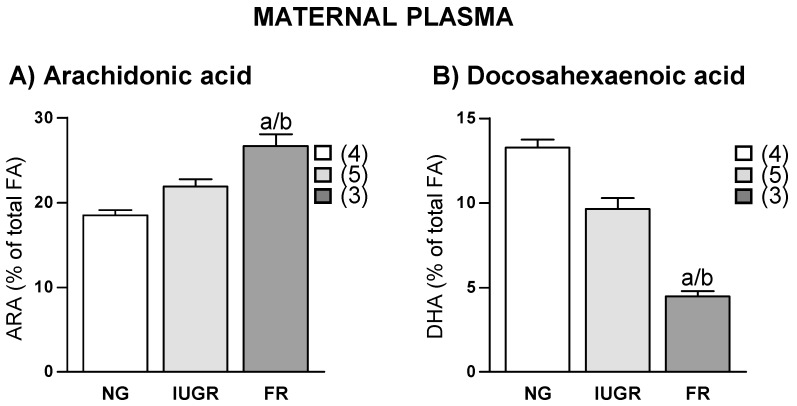
Relationship between fetal outcome and maternal plasma levels of (**A**) Arachidonic acid (ARA) and (**B**) Docosahexaenoic acid (DHA) in experimental diet fed dams. NG, Normal growth; IUGR, Intrauterine growth restriction; and FR, Fetal reabsorption. Data show mean ± SEM. Between parenthesis, the sample size is shown. ANOVA. ^a^ *p* < 0.05 FR versus NG; ^b^ and *p* < 0.05 FR versus IUGR.

CD group had an average of 15 ± 3 pups per litter. We observed that the ED group had three different types of gestation outcome; 4 of the dams had a normal litter size (average 16 ± 3 pups/litter), 5 dams had small litter size (average 7 ± 4 pups/litter) and IUGR, and 3 of them did not deliver any pups. In this last case, we evidenced the presence of fetal reabsorption in the uterine horns. None of the CD dams exhibited small litter size or fetal reabsorption. In ED dams, a relationship between gestation outcome and LCPUFAs was observed; the highest ARA levels were found in dams with fetal reabsorption, followed by those with IUGR and the lowest levels were found in dams with normal fetal growth (Figure 2A). DHA levels followed the opposite pattern (Figure 2B).

**Figure 3:**
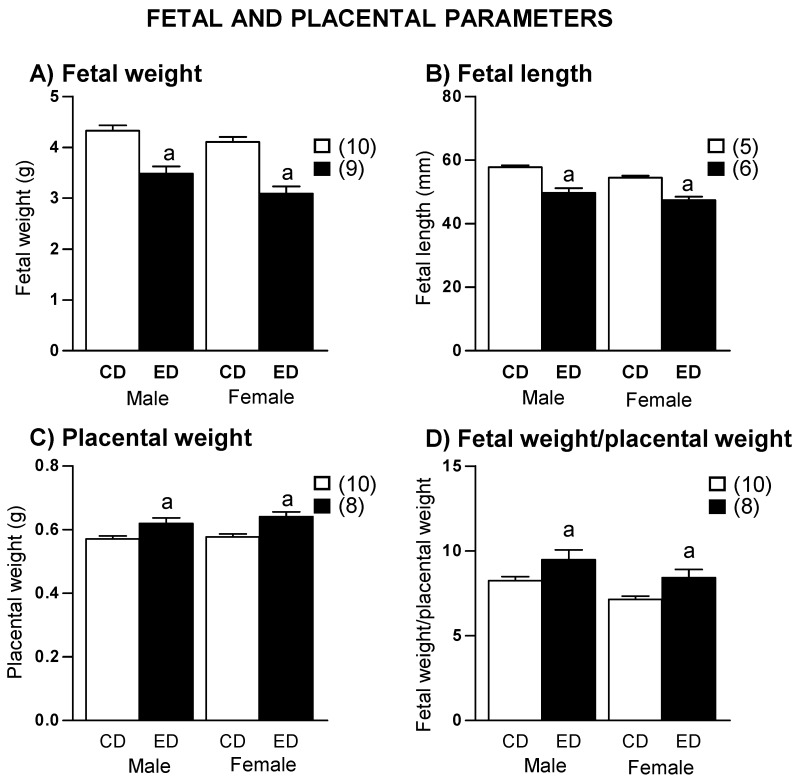
(**A**) Fetal body weight, (**B**) fetal body length, (**C**) placental weight, and (**D**) fetal weight/placental weight in control (CD) and experimental diet (ED) fed groups. Data show mean ± SEM. Between parenthesis, the litter sample size is shown (from each litter 5–6 fetuses of each sex were averaged). Student’s *t* test. ^a^ *p* < 0.01 ED versus CD.

Average fetal body weight and length were significantly smaller in ED compared with CD fetuses, both in males and in females (Figure 3A,B). Fetal crown-rump-length (CRL) and body mass index (BMI) were smaller in ED compared to CD fetuses, both in males and females (Table 1). Considering IUGR individuals below the 3rd percentile, the percentage of IUGR was larger in ED offspring (40.4% of males and 50.0% of females) than in the CD group (2.6% of males and 2.9% of females).

### 2.4. Placenta

**Figure 4:**
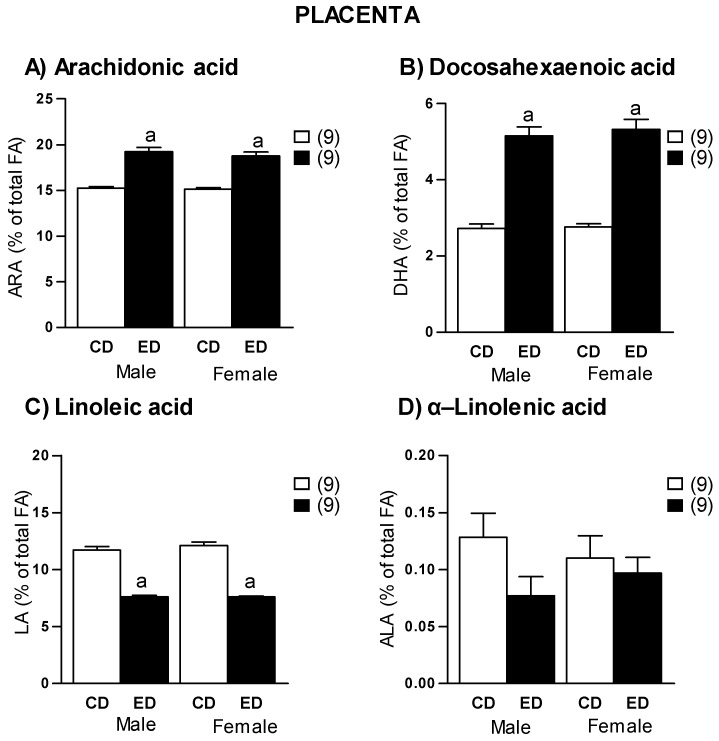
Placental levels of (**A**) Arachidonic acid (ARA), (**B**) docosahexaenoic acid (DHA), (**C**) linoleic acid (LA), and (**D**) α-Linolenic acid (ALA) in dams fed with control diet (CD) and experimental diet (ED), separated by sex. Data show mean ± SEM. Between parenthesis, the litter sample size is shown (from each litter 2–3 placentas of each sex were averaged). Student’s *t* test. ^a^ *p* < 0.001 ED versus CD.

Placental weight was significantly larger in ED compared to CD groups in both sexes (Figure 3C). The relationship fetal body weight/placental weight was significantly smaller in ED rats, both in males and females (Figure 3D). As observed in plasma, placentas from ED group showed significantly higher concentrations of ARA and DHA, compared to CD in both males and females (Figure 4A,B). Regarding the precursors, we found a significantly lower LA in ED group compared to CD in both sexes (Figure 4C), while there were not significant differences in ALA levels between groups (Figure 4D). A significant positive correlation was found between maternal plasma and placental ARA levels (*r* = 0.94, *r*^2^ = 0.87, *p* = 0.0001), a similar positive correlation was found between maternal plasma and placental DHA levels (*r* = 0.79, *r*^2^ = 0.62, *p* = 0.0001).

**Figure 5:**
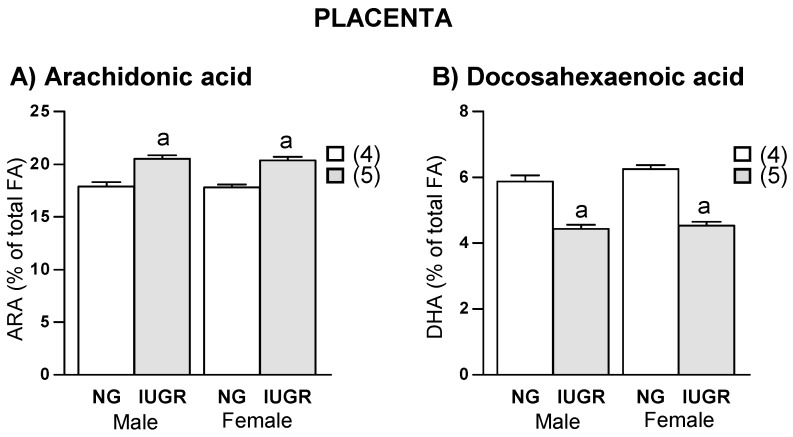
Relationship between fetal growth and placental levels of (**A**) arachidonic acid (ARA) and (**B**) docosahexaenoic acid (DHA) in experimental diet, separated by sex. NG, Normal growth; IUGR, Intrauterine growth restriction. Data show mean ± SEM. Between parenthesis, the litter sample size is shown. ANOVA. ^a^ *p* < 0.001 IUGR versus NG.

**Figure 6:**
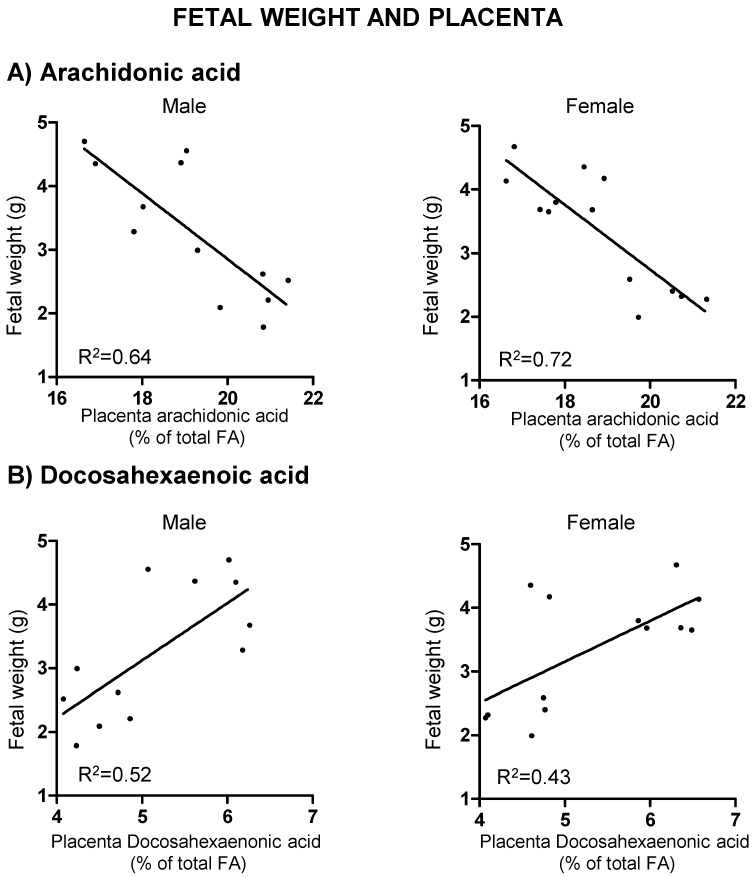
Correlations between male and female fetal weights and placental levels of (**A**) arachidonic acid and (**B**) docosahexaenoic acid in experimental diet fed dams. Linear regression model. Significant interaction is shown as adjusted *R*^2^ values.

In the ED group, placentas from IUGR fetuses exhibited a significantly higher ARA and lower DHA levels compared to those with normal growth; this was observed in both sexes (Figure 5). A negative correlation was found between placental ARA levels and fetal body weight, both in males (*r* = −0.80, *r*^2^ = 0.64, *p* = 0.002) and females (*r* = −0.84, *r*^2^ = 0.72, *p* = 0.001) (Figure 6A). Instead, there was a positive correlation between placental DHA levels and fetal weight, both in males (*r* = 0.72, *r*^2^ = 0.52, *p* = 0.008) and females (*r* = 0.66, *r*^2^ = 0.43, *p* = 0.014) (Figure 6B). In the CD group, no significant correlations were found between LCPUFA and fetal weight. A linear regression model showed that placental ARA and DHA levels explain 56.7% of fetal weight in males and 71.7% of fetal weight in female fetuses.

**Figure 7:**
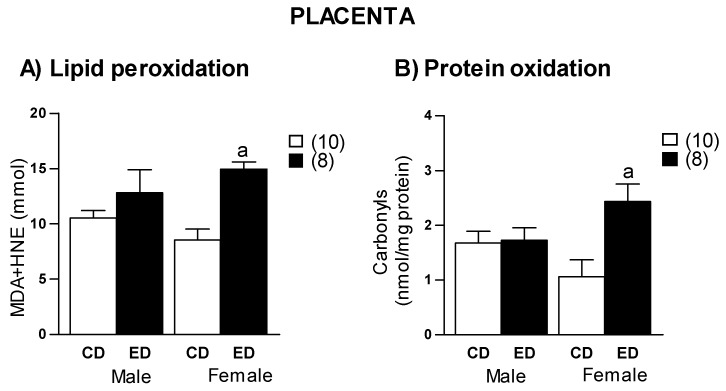
(**A**) Lipid peroxidation and (**B**) protein oxidation levels, in placentas from control diet (CD) and experimental diet (ED), separated by sex. MDA, malondialdehyde; HNE, hydroxynonenal. Data show mean ± SEM. Between parenthesis, the sample size is shown. ANOVA. ^a^ *p* < 0.001 ED versus CD.

Both lipid peroxidation and protein oxidation were significantly higher in female placenta from ED compared to CD group, without significant differences in males (Figure 7). In ED group, a negative correlation between placental lipid peroxidation and fetal weight was found in females (*r* = −0.69, *r*^2^ = 0.47, *p* = 0.05), but not in males (*p* = −0.15; *p* = 0.72). No correlation was found between protein oxidation and fetal weight, either in males (*r* = −0.13; *p* = 0.76) or females (*r* = −0.42; *p* = 0.26). A linear regression model showed that placental lipid peroxidation explains 41% of female fetal weight. In addition, in ED group an association between maternal ARA levels and placenta lipid peroxidation was found (*r* = 0.70, *r*^2^ = 0.49, *p* = 0.01). However, this association was not found in the CD group (*r* = 0.18, *p* = 0.76).

## 3. Discussion

**Figure 8:**
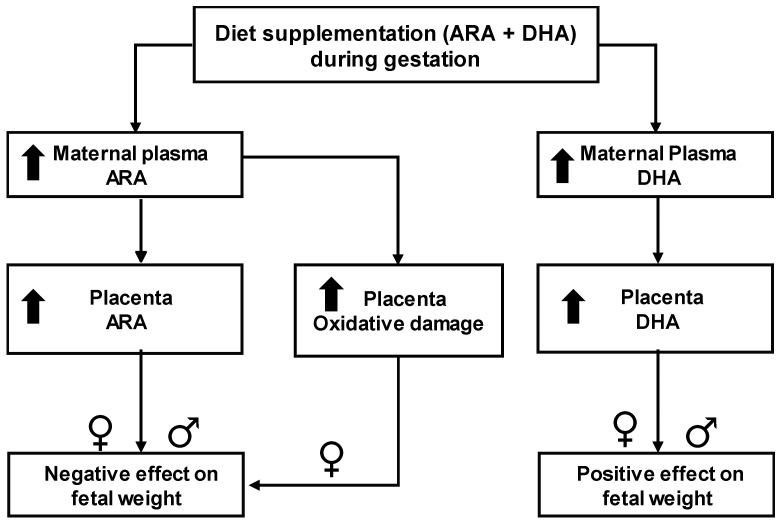
Schematic diagram showing main findings. ARA, arachidonic acid; DHA, docosahexaenoic acid; up-arrows indicate an elevation in response to the dietary intervention.

We aimed to analyze the effects of an experimental rodent diet supplemented with high ARA and DHA content in order to shed light on the controversy regarding the roles of these semi-essential fatty acids on fetal growth. We tried to answer the question if ARA supplementation together with DHA would be beneficial or detrimental, and the relative contribution of each LCPUFA to the response. The main findings of our study are the harmful effects of an excess in maternal ARA on pregnancy outcome and fetal growth. Instead, DHA seems to have beneficial effects on fetal growth, even at high concentrations. We suggest that the deleterious effects of ARA seem to be related to accumulation in the placenta and increased oxidative damage. Our data also suggests that the LCPUFA in maternal plasma and in the placenta are not only dependent on dietary intake, but also on individual maternal metabolism. A summary of the main findings is shown in Figure 8.

### 3.1. Maternal Bioavailability of LCPUFA from the Diet

The main difference between CD and ED diet was the type of fat, soybean or Formulaid™, respectively. The other components were similar in both diets; they were isocaloric and had the same hardness. The source of DHA in ED was microalgae. We chose this source to avoid possible rejection of the food by the rats due to fish smell. In fact, we demonstrated that rats ate the same amount of CD and ED chow during gestation. The analysis of LCPUFA by GC/MS revealed that both ARA and DHA were absent in CD, but it had the precursors, LA and ALA.

We first aimed to analyze the bioavailability in maternal plasma of dietary LCPUFA. DHA and ARA from the diet were absorbed, as shown by the higher concentration found in plasma from ED-fed dams. We provide data only of the phospholipid fraction, which has a lower turnover than plasma triacylglycerols and better reflects the fatty acid intake [22], but we had similar results in the other fractions analyzed. The precursors (LA and ALA) were also incorporated from the diet, as shown by the similar proportion in the diet and in the maternal plasma. LA and ALA were available for biosynthesis, evidenced by the presence of DHA and ARA in rats fed with CD chow, in which these FA were absent.

### 3.2. Effect of Dietary Intervention on Gestation

The experimental diet restricted fetal growth, evidenced by a lower weight and length of the fetuses at G20. This effect was not related to lack of nutrients, since CD and ED chow had the same caloric content, similar macronutrient proportion and CD and ED rats ate the same amount of food. Besides, similar glucose and protein levels were found in the plasma of dams fed with either diet. The deleterious effect of ED was specific to fetal growth and did not affect maternal weight gain, evidenced by the similar dam weight without conceptus at the end of gestation (G20).

We also observed different gestational outcomes in response to ED. While some ED-fed rats had litters of normal weight, length, and number, other dams had offspring with signs of IUGR and some rats even had complete fetal reabsorption, evidenced in the uterine horns. We found important to define what we considered IUGR in rats. This is relevant considering the relationship between fetal growth restriction and disease programming [23]. In this study, we considered growth restriction a fetal weight below the 3rd percentile, which is a common criterion in humans based only on weight [24]. Another criterion used in humans to define IUGR is a birth weight below the 10th percentile if hemodynamic compromise is present [25]. We did not use this standard since we did not evaluate uterine flow. Our data showed that ED-fed dams had a large proportion of IUGR fetuses (over 40%). In CD rats less than 2.5% of the animals, fetal growth was restricted. These data were obtained in fetus from several dams and cannot be attributed to a litter effect, which is important in studies on the effects of gestational interventions [26]. It would be desirable to confirm if this rate of IUGR is similar in mice and in other rat strains.

We explored whether the deleterious effects of the experimental diet on fetal growth could be attributed to ARA or DHA. Our data point out at an excess ARA, as shown by the association between placental ARA levels and growth restriction. Instead, DHA exhibited a positive relationship with fetal growth. It is interesting to note that, with the same diet, the dams showed different plasma concentrations of LCPUFA, which could be attributed to individual differences in the enzymatic systems implicated in their metabolism. The fact that there was a negative correlation between ARA and DHA, both in plasma and placenta, suggests that there was an interaction between both LCPUFA. We suggest that an excess of ARA might be interfering with DHA metabolism since they share some enzymatic systems. A similar interaction has been previously described in dams exposed to diet supplemented with fish oil, where excess DHA has been proposed to participate in the reduction of ARA through decreased expression of Δ6-desaturase [15].

### 3.3. Role of Placenta

Our data indicate that the placenta plays a key role in the deleterious response to the diet. Placental weight was larger in rats exposed to the ED and a close parallelism was found between DHA and ARA levels in maternal plasma and in the placenta. We suggest that the LCPUFA may be accumulating in the placenta, being used for placental growth and may not be available for the fetus. Similar results have been described in other studies using LCPUFA enriched diets, where higher ARA concentrations decrease fetal growth while they increased placental weight [27]. In ED-fed rats fetal weight, the placental weight ratio was lower compared to CD rats, suggesting a poorer efficiency of the placenta to supply sufficient nutrients to meet the fetal growth demand. Similar findings have been reported in rodent models of IUGR [28]. Furthermore, human pregnancies with IUGR exhibit an increased n-6 and n-3 FA in the placenta and it has been suggested that, if LCPUFA are preferentially routed towards placental storage, availability for the fetus may be reduced [29]. Our data show a lower content of the ARA precursor, LA, in the placenta from ED fed dams. This may suggest that ARA accumulation may also be due to local synthesis.

Pregnancy is often described as a state of controlled, physiological systemic inflammation with high levels of ROS, particularly in early stages [30]. However, in excess, oxidative stress and inflammation lead to adverse pregnancy outcome [31]. We found larger levels of protein and lipid damage in female placenta from ED-fed dams, suggesting that placenta oxidative stress might be implicated in the deleterious response to the diet. A positive relationship between maternal ARA levels and placenta lipid peroxidation was found. However, this relationship was not observed with DHA. These data indicate an implication of ARA in the oxidative processes, probably through an inflammatory environment, since ARA-derived eicosanoids contribute to inflammation [17]. Pro-inflammatory cytokines are generated in response to ROS and an increased inflammatory environment further stimulates ROS generation [32]. Furthermore, there is also evidence that placental ROS can inhibit fetal growth [4]. Our data let us conclude that high ARA intake may be accumulated in the placenta, where an excess may compromise fetal growth through an oxidative stress/inflammation-related mechanism.

### 3.4. Effect of Sex

Sex differences in response to adverse uterine environment are well recognized. However, the majority of studies [23], including ours in a rat model of gestational undernutrition, show that in the short term, females are better adapted to adverse fetal environments [33,34,35]. In the present study, both sexes seem to be similarly affected by the diet in terms of growth. However, the contribution of oxidative stress to IUGR seems to be sex dependent, with a worse adaptation of females. The placenta plays a key role in the sexual dimorphic response to fetal stressors. On one hand, it is the physical and metabolic interface between maternal environment and the fetus and it bears the same sex of the fetus [36]. Besides, IUGR is associated with placental oxidative stress in response to maternal exposure to adverse factors, such as under nutrition, excess glucocorticoids, or toxic substances [35]. It has been proposed that murine placental responses to maternal diets exhibit a pronounced sexual dimorphism, with the female placenta being more sensitive to nutritional interventions with a larger change in gene expression [37]. Our data also indicate that female placenta has a larger susceptibility to the excess in dietary ARA, evidenced by the higher oxidative damage to lipids and proteins in response to the ED and the negative relationship between placental lipid peroxidation and female fetal weight.

In conclusion, the present data demonstrate a relationship between excess ARA in diet during gestation and fetal growth restriction, while DHA seems to have favorable effects on fetal growth. Our data points out at the importance of fat content in the diet during pregnancy and the correct use of LCPUFA supplements in the context of pregnancy. This study has been conducted in rodents and extrapolation to the human condition should be done with caution.

## 4. Methods

### 4.1. Diet Preparation

The experimental diet (ED) was prepared specifically for the study by TestDiet^®^ Company (Richmond, IN, USA) based on the composition of EuroRodent Diet 22 (Control diet, CD), replacing the soybean oil by Formulaid™ (2:1) (DSM Nutritional Products, Basel, Switzerland), as energy source. Formulaid™ (2:1) is an oil derived from fungi and microalgae in the form of triglycerides, containing, among other fatty acids, ARA (20:4n-6) and DHA (22:6n-3) in the proportion of 2:1. It also contains ascorbyl palmitate (250 ppm) and tocopherols (250–500 ppm) as antioxidants. During diet preparation, the oil was protected from exposure to oxygen, it was prepared at low temperature and it was stored frozen until use.

CD and ED were isoenergetic (4.03 kcal/g) and they did not differ in protein, carbohydrate, total fat, and vitamin content (Table 2).

The fatty acid composition of CD and ED was analyzed by gas chromatography/mass spectrophotometry (GC/MS), as previously reported by Carnielli [38]. ARA and DHA were not detectable in CD, while ED had both LC-PUFAs (ARA = 13.5%; DHA = 6.3%; expressed as a percentage of the total FA composition). The precursors, LA and ALA, were present in both diets (Table 3).

### 4.2. Experimental Animals

Experiments were performed in Sprague Dawley rats from the colony maintained at the Animal House facility of the Universidad Autónoma de Madrid. All experimental procedures were approved by the Ethics Review Board of Universidad Autónoma de Madrid (Animal Wellbeing Committee, 15 April 2015). These procedures also conformed to the Guidelines for the Care and Use of Laboratory Animals (NIH publication No. 85-23, revised in 1996), the Spanish legislation (RD 1201/2005) and the regulations of the local Government (Comunidad Autónoma de Madrid; Experimental Protocol Number 199/15).

Rats were housed in buckets 36.5/21.5/18.5 cm (length/width/height) on aspen wood bedding and maintained under controlled conditions (temperature 22 °C, relative humidity 40%, and 12/12 light/dark photoperiod) and the welfare of the animals was monitored by staff at least once a day.

### 4.3. Experimental Procedures

12 weeks old rats were mated and day 1 of gestation was determined in the dams by observation of sperm in the vaginal smear. Thereafter, they were weighted and randomly allocated to one of the groups CD (315.6 ± 24.1 g; *n* = 11) or ED (323.1 ± 12.1 g; *n* = 12), for the entire gestational period. In both groups, food and water were provided ad libitum and the diet was changed in the hoppers every day. A group of experiments was designed to monitor the amount of diet that the dams eat daily.

The experiments were performed at gestational day 20 (G20). The dams were anaesthetized in a CO_2_ atmosphere and a blood sample was obtained, in tubes containing heparin, by cardiac puncture. The blood was centrifuged at 900× *g* for 10 min at 4 °C to obtain plasma which was aliquoted and stored at −80 °C until use. The dam was killed by exsanguination and the uterine horns were carefully dissected. The individual fetus and placenta pairs were obtained.

The fetuses were sexed; each fetus and its placenta were weighted with a precision scale and the fetus length was measured with a digital caliper (Conecta, Nessler). Fetal body weight below the 3rd percentile was established as criteria of IUGR.

Placental tissue was homogenized (100 mg of tissue in 1 mL of NaCl 0.9% solution with protease inhibitor cocktail) and stored at −80 °C for future analysis.

Bradford assay (Bio-Rad) and a digital glucometer (Accu-check Aviva, Roche) were used to assess maternal plasma protein and glucose concentrations, respectively.

Phospholipids and total FA were assessed in maternal plasma and placentas by GC/MS as previously reported [38]. Data are expressed as a percentage of the total FA composition.

Oxidative damage to lipids in the placenta was assessed by a lipid Hydroperoxide Assay Kit (LPO, Bioquochem, Gijón, Spain), which analyses malondialdehyde (MDA) + hydroxynonenal (HNE) concentrations. The experiments were performed according to the manufacturer’s instructions and the data were expressed as μmol.

Oxidative damage to proteins was assessed by 2,4 dinitrophenylhydrazine-based assay that detects protein carbonyls [39], adapted to microplate reader [40], using extinction coefficient of 2,4-dinitrophenylhydrazine (ε 22,000 M/cm). The absorbance was measured at 595 nm in a Synergy HT Multi-Mode Microplate Reader (Synergy^TM^-HT, BioTek, Winooski, VT, USA) and the data were expressed as nmol/mg protein.

### 4.4. Statistical Analysis

All the variables analyzed followed a normal distribution (Kolmogorov-Smirnov test). Data are expressed as mean ± SEM, considering the litter as sample number. From each dam data of males and females were averaged. Data were analyzed by Student’s t test or ANOVA, using a linear regression model, with maternal diet and fetal sex (where applicable) as co-variables. Correlations were evaluated by Pearson’s p (*p*). The probability value (*p*) at which differences were considered significant was *p* < 0.05. Statistical analysis was performed with SPSS 22.0 (IBM, Corp., Armonk, NY, USA).

## Figures and Tables

**Table 1:** Fetal anthropometric parameters.

	Males		Females	
	CD	ED	CD	ED
CRL (mm)	44.7 ± 0.4	42.7 ± 0.6 ^a^	42.7 ± 0.5	40.4 ± 0.6 ^a^
BMI (g/cm^2^)	0.254 ± 0.004	0.218 ± 0.004 ^b^	0.258 ± 0.004	0.204 ± 0.004 ^b^

CD, Control diet; ED, Experimental diet; CRL, crown-rump length; BMI, body mass index; ^a^ *p* < 0.05, and ^b^ *p* < 0.001 with respect to sex-matched CD.

**Table 2:** Composition of the diets.

Diet Composition	CD	ED
Total digestible nutrients (%)	77.8	73.2
Calories provided by protein (%)	25.9	25.7
Calories provided by CHO (%)	64.8	62.9
Calories provided by fat (%)	9.3	10.7
Protein content (%)	22.0	22.0
CHO (nitrogen free extract) content (%)	55.0	53.9
Total fat content (%)	4.4	4.4
Ash content (%)	5.4	5.4
Fiber maximum content (%)	4.1	4.3
Fat source	Soybean oil	Formulaid™ 2:1

Composition was provided by manufacturer. Moisture content is assumed to be 10.0% for the purpose of calculations. CD, control diet; ED, experimental diet; and CHO, carbohydrates.

**Table 3:** Fatty Acid composition of the diets.

Fatty Acids (%)	ED	CD
Lauric Acid (12:0)	1.3	-
Myristic Acid (14:0)	3.1	0.1
Palmitic Acid (16:0)	14.2	16.3
Palmitoleic Acid (16:1w7)	0.5	0.2
Stearic Acid (18:0)	4.5	2.7
Oleic Acid (18:1w9)	20.8	20.4
Linoleic Acid (18:2w6)	27.5	53.5
Linolenic Acid (18:3w6)	1.0	-
Alpha Linoleic Acid (18:3w3)	3.0	4.6
Arachidic Acid (20:0)	0.4	0.3
Gondoic Acid (20:1w9)	0.2	0.3
Eicosadienoic Acid (20:2w6)	0.2	-
Eicosatrienoic Acid (20:3w6)	0.9	-
Arachidonic Acid (20:4w6)	13.5	-
Eicosapentaenoic Acid (20:5w3)	0.1	-
Behenic Acid (22:0)	0.5	-
Docosahexaenoic Acid (22:6w3)	6.3	-
Lignoceric Acid (24:0)	0.4	-

CD, control diet; ED, experimental diet.
